# Overexpression of p53R2 is associated with poor prognosis in lung sarcomatoid carcinoma

**DOI:** 10.1186/s12885-017-3811-6

**Published:** 2017-12-15

**Authors:** Jiewei Chen, Yongbo Xiao, Xiaoyan Cai, Jun Liu, Keming Chen, Xinke Zhang

**Affiliations:** 1 Department of Pathology, Sun Yat-Sen University Cancer Center, Guangzhou, 510060 China; 2 Department of Pathology, Taishan People’s Hospital, Taishan, Guangdong 529200 China; 3 Sun Yat-sen University Cancer Center, State Key Laboratory of Oncology in South China, Collaborative Innovation Center for Cancer Medicine, Guangzhou, China

**Keywords:** Lung sarcomatoid carcinoma, p53R2, Immunohistochemistry, Prognosis

## Abstract

**Background:**

This study aimmed to evaluate the expression of p53-inducible RR small subunit 2 homologue (p53R2) in Lung sarcomatoid carcinoma (LSC) and its association with clinicopathological parameters and prognosis.

**Methods:**

In this study, clinicopathological factors and prognostic significance of the expression of p53R2 was investigated by immunohistochemistry (IHC) in 100 cases of LSC.

**Results:**

The results showed that the expression of p53R2 was significantly correlated with clinical stage (*P*<0.05). But there was no statistically correlation with gender, age, smoking, tumor size, pT stage, pN stage, pM stage, therapy and relapse. Kaplan-Meier analysis revealed that the expression of p53R2, clinical stage, pT stage, pN stage, pM stage and tumor size were closely related to patients’ survival, and the analysis also revealed that patients with low expression of p53R2 had a longer overall survival than that with high expression (Mean overall survival: 84.8 months vs. 34.7 months, *P*<0.05). Further multivariate analysis indicated that the expression of p53R2 was identified as an independent prognostic factor in the prediction of the overall survival for patients with LSC (HR = 3.217, *P<*0.05).

**Conclusions:**

The expression of p53R2 was inversely associated with the proliferation and progression of LSC, and the results indicated that the high expression of p53R2 was an independent factor for unfavorable prognosis of patients with LSC.

## Background

2016 Cancer statistics show that morbidity and mortality of lung cancer rank second, and is obviously threatening human health in the United States [1]. Lung sarcomatoid carcinoma (LSC) is a subtype of non-small cell lung cancer (NSCLCs) with histology containing sarcoma or sarcomatoid differentiation. The incidence of LSC represent appropriately from 0.1% to 0.4% in lung malignancies [2], and approximately 2.35% of NSCLCs [3]. Although the rare proportion is occurred, nearly 2000 cases were diagnosed as LSC per year in the United States [4]. LSC is characterized by highly malignant and easy to relapse [5]. The five-year survival rate is approximately 24.5%, which is significantly lower than other types of NSCLCs [6]. So far, the efficacy of systemic chemotherapy has not been clearly identified in patients with LSC, commonly chemotherapy regimens applied for NSCLC did not work well [7]. Therefore, it is urgently necessary to explore novel biomarkers for predicting clinical outcomes and find new effective therapeutic strategies for LSC.

P53R2, also known as RRM2B, is primarily identified as a ribonucleotide reductase small subunit in the colon cancer cell line by Japanese researchers [8], and is a downstream target gene of the p53 gene consisting of 9 exons and 1 intron that binds to the p53 sequence, could provides dNTPs for DNA synthesis and repair in the G1 and G2 phases of the cell cycle [9, 10]. Previous studies have shown that expression of p53R2 is increased in the presence of radiation or genotoxicity [11],p53R2 has dual role for tumor regulation, which include tumor suppression depending on p21 signal pathway for promoting cell apoptosis and inhibiting cell proliferation, however, tumor progression is carried out through its anti-reactive oxygen species potential and resistance to therapy [12, 13].The expression of p53R2 has been investigated in different human tumors, but the results of association between the expression level of p53R2 and patients’ prognosis remains to be controversial. A study revealed that high expression of p53R2 was significantly correlated with a better survival of patients with late-stage colorectal cancer [14], another literature demonstrated that high expression of p53R2 is associated with tumor progression in patients with esophageal squamous cell carcinoma [15]. However, there are no relevant reports on the prognostic value of p53R2 in LSC. In this study, we investigated the expression status of p53R2 protein in LSC by immunohistochemistry.

## Methods

### Patients and tissue specimens

Clincopathological data of the retrospective analysis for 100 patients with lung sarcomatoid carcinoma who had pneumonectomy and/or lymphadenectomy were collected from the Department of Pathology, Sun Yat-Sen University Cancer Center (February 2000 to March 2016) by removing the patients with neoadjuvant therapy. Eight of 100 cases had the synchronous metastases. All cases were diagnosed according to the WHO classification criteria in 2004, 2002 US Joint Commission and the International Joint Cancer TNM Classification System, and the study was approved by the Sun Yat-Sen University Cancer Center Medical Ethics Committee.

### Immunohistochemistry (IHC)

The immunohistochemical expression of p53R2 was evaluated according to standard EnVision™ procedure in a tissue microarray (TMA). 3-μm Paraffin blocks sections was used to perform IHC. The slides were deparaffinized with xylene and rehydrated through graded alcohol, and then they were immersed in citrate buffer for antigen retrieval by pressure cooking about 3 min. Subsequently, the TMA slides were incubated with antibody p53R2 (abcam, ab8105, dilution 1: 400) at 37 °C for 50 min. For blocking the endogenous peroxidase activity, the slides were placed in 3% hydrogen peroxide for 10 min, and were sequentially incubated with secondary antibody (DAKO, K5007) at 37 °C in the incubator for 30 min. Then, they were stained with 3,3-diaminobenzidine(DAB). Finally, the slides were counterstained with hematoxylin, dehydrated and mounted. Positive and negative controls were obtained.

### IHC evaluation

The assessment of p53R2 expression was performed by two independent pathologists. The number of positively stained tumor cells is defined as a percentage (%), and the intensity of staining is evaluated as (“-”, “1+”, “2+”, “3+”); Finally, each intensity multiplied by the percentage of the corresponding positive cells to obtain the scores of each sample.

### Selection of cutoff score

An receiver operating characteristic (ROC) curve is performed according to varying cut-offs for sensitivity and corresponding 1-specificity, the suitable cut-off value could be identified by ROC curve analysis [16], the sensitivity and specificity for each clinicopathological factor was plotted for the score of P53R2 in our study, and generating different ROC curves. The score was chosen as the cutoff value with both maximum sensitivity and specificity.

### Statistical analysis

Statistical analysis was performed using SPSS16.0. Correlation between p53R2 protein expression and clinicopathological parameters in patients with LSC was analyzed by Chi-square test. The survival analysis of LSC patients was evaluated by the Kaplan-Meier method with log-rank test. Multivariate analyses were performed using Cox proportional hazard model. All *P* values were reported by two-sided analyses and *P* < 0.05 stands for the statistical significance level.

## Results

### Patients’ characteristics

**Fig. 1:**
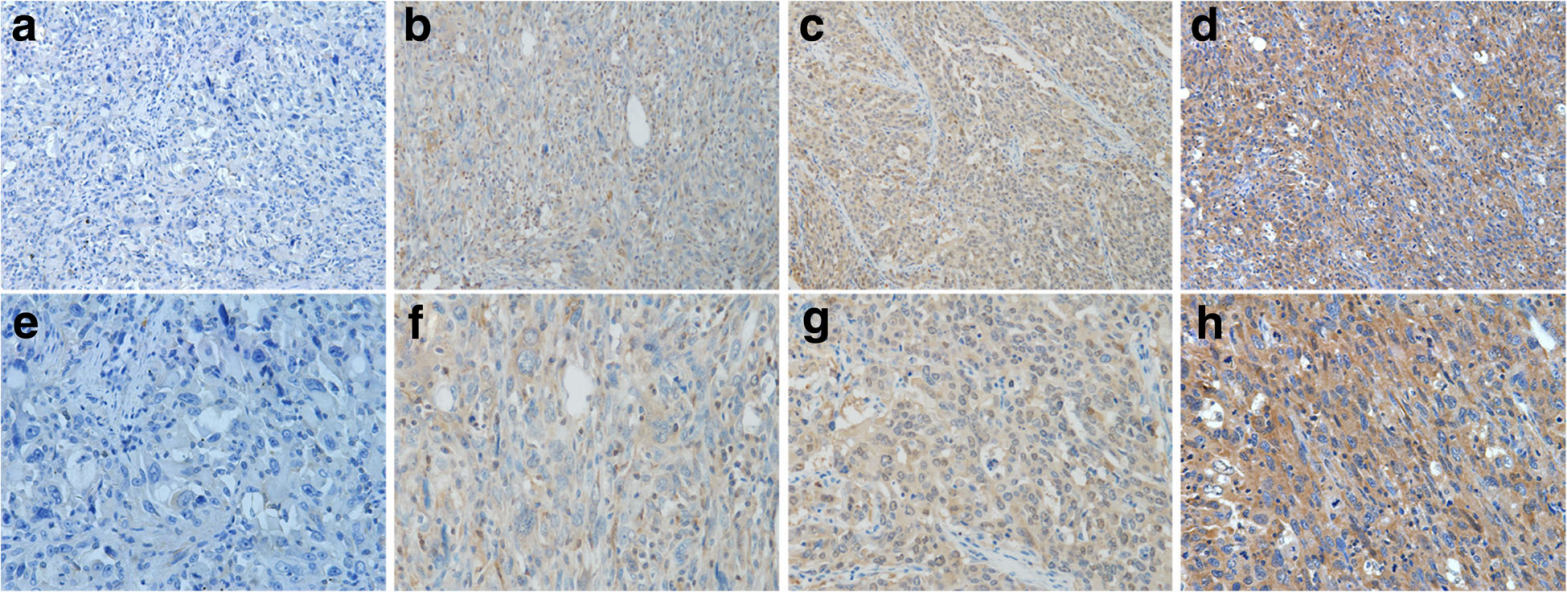
Expression of p53R2 in Lung sarcomatoid carcinoma. **a**, **e** (Negative expression); **b**, **f** (Low expression); **c**, **g** (moderate expression); **d**, **h** (Strong expression of p53R2)

The clinicopathological characteristics of LSC patients were detailed in Table 1. This LSC cohort consisted of 87 (87.0%) men and 13 (13.0%) women with mean age of 57 years. Average follow-up period was 26.2 months (median, 27.4 months; range, 1.0 to 129.0 months). 55 patients (55.0%) were diagnosed at late stages (III and IV), and the other 45 patients (45.0%) were at early stages (I and II). Immunohistochemical results showed that p53R2 protein was mainly located in the cytoplasm of LSC (Fig. 1).

**Table 1:** Correlation between the clinicopathologic variables and expression of p53R2 in LSC

Variable	Expression of p53R2			
	All cases	Low expression	High expression	*P* value^a^
Age (years)^b^				0.084
≤ 57	47	24 (51.1%)	23 (48.9%)	
> 57	53	18 (34.0%)	35 (66.0%)	
Gender				0.782
Male	87	37 (42.5%)	50 (57.5%)	
Female	13	5 (38.5%)	8 (61.5%)	
Tumor size^c^				0.928
≤ 4.5	59	25 (42.4%)	34 (57.6%)	
> 4.5	41	17 (41.5%)	24 (58.5%)	
Smoking				0.144
Yes	76	35 (46.1%)	41 (53.9%)	
No	24	7 (29.2%)	17 (70.8%)	
T stage				0.155
T1 And T2	56	27 (48.2%)	29 (51.8%)	
T3 And T4	44	15 (34.1%)	29 (65.9%)	
Lymph node				0.638
N0	52	23 (44.2%)	29 (55.8%)	
N1,N2 And N3	48	19 (39.6%)	29 (60.4%)	
M stage				0.310
M0	92	40 (43.5%)	52 (56.5%)	
M1	8	2 (25.0%)	6 (75.0%)	
Clinical stage				0.038
I-II	45	24 (53.3%)	21 (46.7%)	
III-IV	55	18 (32.7%)	37 (67.3%)	
Therapy^d^				0.996
Therapy1	76	32 (42.1%)	44 (57.9%)	
Therapy2	19	8 (42.1%)	11 (57.9%)	
Therapy3	5	2 (40.0%)	3 (60.0%)	
Relapse				0.479
Yes	30	11 (36.7%)	19 (63.3%)	
No	70	31 (44.3%)	39 (55.7%)	

^a^Chi-square test

^b^Mean age

^c^Mean tumor size

^d^Therapy:Therapy1 is Pneumonectomy and lymphadenectomy,Therapy2 is Pneumonectomy;Therapy3 Pneumonectomy and radiotherapy and chemoradiotherapy

### Selection of cutoff value for p53R2 expression

**Fig. 2:**
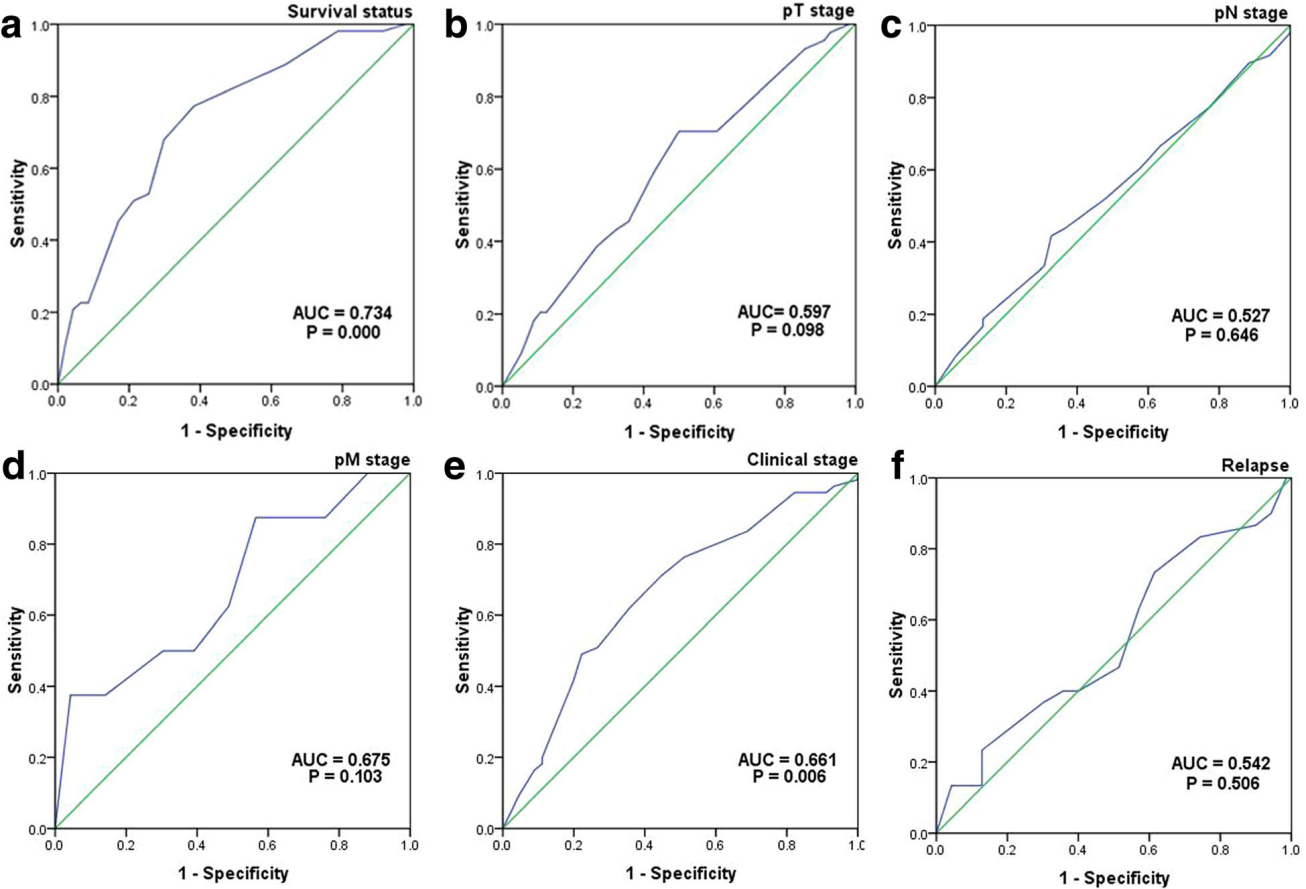
ROC curve analysis was employed to determine the cutoff value for high expression of p53R2 in Lung sarcomatoid carcinoma. The sensitivity and specificity for each outcome were plotted: Survival status (*P* = 0.000, **a**), pT stage (*P* = 0.098, **b**), pN stage (*P* = 0.646, **c**), pM stage (*P* = 0.103, **d**), Clinical stage (*P* = 0.006, **e**), and Relaspe (*P* = 0.542, **f**)

To choose a suitable cutoff score of p53R2 for further analysis, each clinicopathological parameter is used to analyze in the ROC curve, respectively (Fig. 2), samples with score more than or equal to the obtained cutoff value were seen as high expression of p53R2. According to this method, we found that the survival state is the optimal clinicopathological factor, as it was shown in Fig. 2. On the basis of this outcome, the score of 110 was defined as the optimal cutoff value for p53R2 expression by the survival state for survival analysis, the sensitivity and specificity were 0.774 and 0.609, *P* < 0.001 (Fig. 2).

### Association of p53R2 expression with LSC patients’ Clinicopathological features

The rates of high and low expression of p53R2 in LSC about several clinicopathological features were detailed in Table 1. Chi-square test showed that the high expression of p53R2 protein was significantly correlated with clinical stage (*P* = 0.038); however, there were not significantly correlated with other clinical parameters (Sex, age, smoking or not, tumor size, T stage, N stage, M stage, treatment, recurrence, etc.) (*P* > 0.05) (Table 1).

### Relationship between p53R2 expression and LSC patients’ survival

**Fig. 3:**
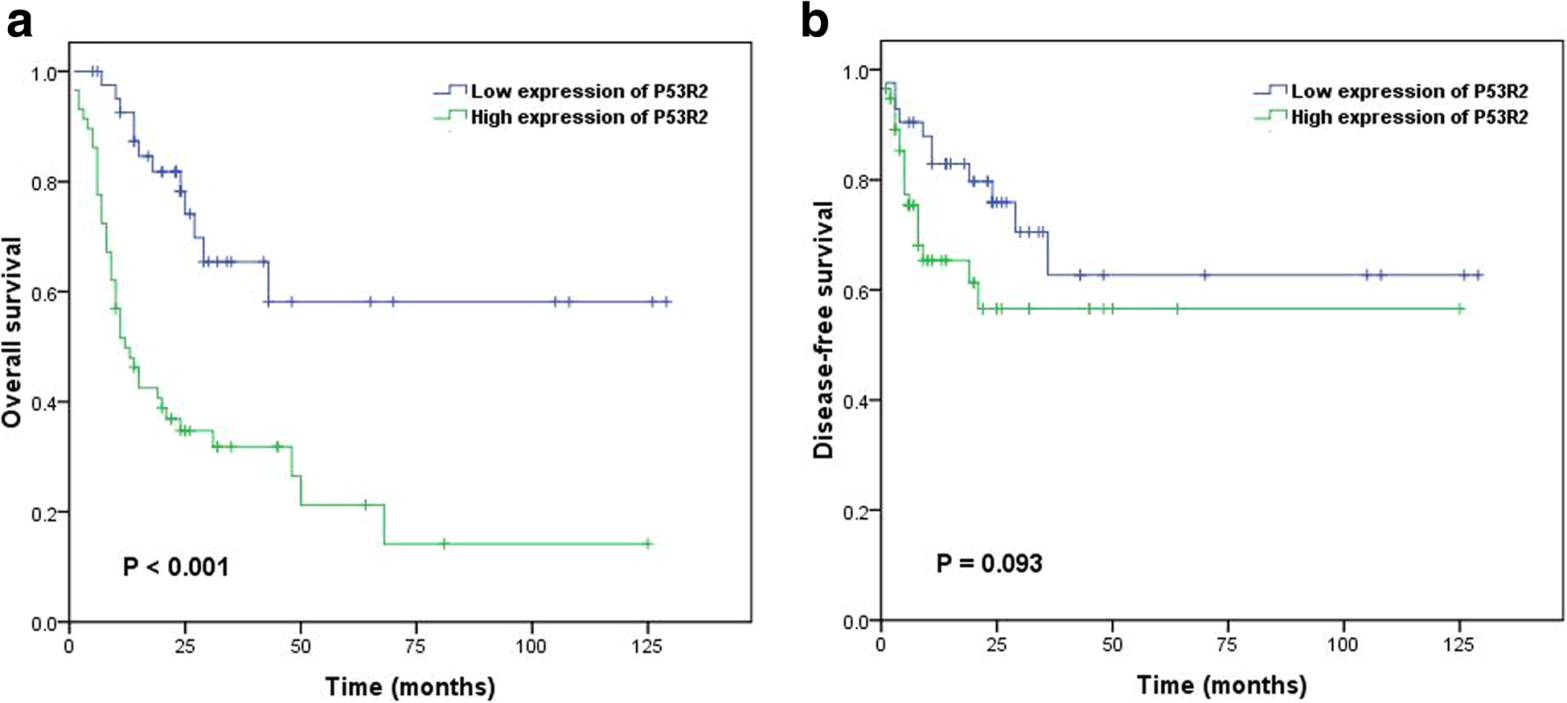
Survival curves of 100 LSC patients for overall survival (**a**) and disease-free survival (**b**)

In this study, we firstly evaluated the impact of clinicopathological prognostic factors, (i.e., Tumor size, T classification, N classification, distant metastasis, clinical stage) on prognosis with LSC patients by univariate analysis (*P* < 0.05, Table 2). It demonstrated that patients with high expression of p53R2 protein were closely associated with unfavorable overall survival (*P* = 0.000, Table 2, Fig. 3a) and were not significantly associated with disease-free survival (*P* = 0.093, Fig. 3b) in 100 patients with LSC. The risk factors of univariate analysis were introduced into COX risk regression model for multivariate analysis, The results showed that clinical stage (*P* = 0.000) and p53R2 protein expression (*P* = 0.000) could be used as independent prognostic factors for LSC patients’ overall survival (HR: 3.217, CI:1.675–6.180; *P* = 0.000, Revised Table 3).

**Table 2:** Univariate analysis of clinicopathologic variables in 100 patients with LSC (log-rank test)

Variable	All cases	Mean survival(months)	Median survival(months)	*P* value^a^
Age (years)^b^				0.439
≤ 57	47	47.8	31.0	
>57	53	55.4	18.0	
Gender				0.538
Female	13	37.4	27.0	
Male	87	55.2	25.0	
Smoking				0.078
Yes	76	59.4	43	
No	24	25.1	15.0	
Tumor size^c^				0.032
≤ 4.5 cm	59	65.8	48.0	
>4.5 cm	41	39.5	18.0	
T stage				0.033
T1 And T2	56	63.2	48.0	
T3 And T4	44	44.7	14.0	
Lymph node				0.000
N0	52	74.7	68.0	
N1,N2 And N3	48	31.4	14.0	
M stage				0.000
M0	92	59.9	43.0	
M1	8	12.1	8.0	
Therapy^d^				0.103
Therapy1	76	62.2	48.0	
Therapy2	19	28.9	21.0	
Therapy3	5	19.0	24.0	
Relapse				0.713
Yes	30	36.4	24.0	
No	70	58.9	43.0	
Clinical stage				0.000
I-II	45	89.8	NR	
III-IV	55	19.5	11.0	
p53R2				0.000
Low expression	42	84.8	NR	
High expression	58	34.7	12.0	

^a^Chi-square test

^b^Mean age

^c^Mean tumor size

^d^Therapy:Therapy1 is Pneumonectomy and lymphadenectomy, Therapy2 is Pneumonectomy;Therapy3 Pneumonectomy and radiotherapy and chemoradiotherapy

**Table 3:** Multivariate analysis of different prognostic factors in 100 patients with LSC

Variable	Hazards ratio	95% CI	*P* value
Tumor size (>4.5 cm vs. ≤4.5 cm)	1.561	0.899–2.708	0.114
Clinical stage (III-IV vs. I-II)	5.160	2.589–10.282	0.000
Expression of p53R2 (High vs. Low)	3.217	1.675–6.180	0.000

Further analysis showed that 6 of 42 patients with low expression of p53R2 and 0 of 58 patients with high expression of p53R2 survived more than 5 years. The average overall survival time of 34.7 months in LSC patients with high expression of p53R2 protein was significantly lower than that of low expression group (84.8 months), suggesting the patients with high expression of p53R2 had worse prognosis than those with low expression of p53R2.

## Discussion

P53R2 is a small subunit of human nucleotide reductase, which is closely related to DNA repair, mtDNA synthesis, blocking cell cycle and maintaining mitochondrial homeostasis [17]. The expression of p53R2 protein is associated with drug sensitivity and tumor invasiveness, suggesting that it can be a potential target for anticancer drugs [18].

In this study, we first used immunohistochemical method to detect the expression of p53R2 protein in LSC tissues. We found that expression of p53R2 is mainly located in the cytoplasm, due to the location of p53R2 protein is affected by a various of factors, a study showed that redistribution of p53R2 protein occurs in response to signals that initiate DNA replication from the cytoplasm to the nucleus [19]. Therefore, we speculated that the possible reason is that p53R2 protein did not exert its role translocating into the nucleus for DNA repair and DNA synthesis during the S phase when R2 subunit is available, in addition, p53R2, R2 binding to p53 located in the cytoplasm in quiescent cells, Which is consistent with prior studies [20, 21]. Chi-square test analysis revealed that p53R2 protein expression and clinical stage has a significantly positive correlation, suggesting that p53R2 protein expression and the occurrence and development of LSC is closely related. Hsu NY studies have shown that p53R2 protein is closely related to the differentiation, stage and lymph node metastasis of NSCLCs and plays an important role in the early stages of recurrence [22],Okumura’s study revealed that p53R2 protein expression was associated with lymph node metastasis, depth of invasion, and clinical stage of esophageal squamous cell carcinoma. [15],The expression of p53R2 was also correlated with tumor size, local lymph node metastasis and histological classification [23], Similarly, the study of Shigeto Matsushita revealed that the expression of p53R2 protein was associated with the depth of invasion and clinical stage of melanoma [24]. All of these studies suggested that p53R2 protein is closely related to the development of tumors, which is agreement with our study.

The relationship between the expression of p53R2 protein and the prognosis of patients with LSC has not been reported. In this study, Kaplan-Meier survival analysis revealed that p53R2 protein expression, tumor size, clinical stage, T stage, N stage and M stage were the prognostic factors for LSC patients(*P*<0.05). Overexpression of p53R2 protein group of patients with LSC, the average overall survival time of 34.7 months, is far lower than that with low expression of p53R2 with overalls survival time of 84.8 month. Cox multivariate analysis revealed that p53R2 protein expression, N stage and M stage could be used as an independent prognostic factor for assessing survival time of LSC patients, which is consistent with Hiroshi Okumura et al. in NSCLCs, esophageal squamous cell carcinoma, oral squamous cell carcinoma and melanoma, and the prognosis is poor in patients with overexpression of p53R2 protein. [15, 22–24]. Souichi Yanamoto’s study showed that p53R2 promotes tongue cancer invasion through E-cadherin/β-catenin pathway [25]. P53R2 is closely related to tumorigenesis, overexpression of p53R2 could cause myelodysplasia syndrome and acute myeloid leukemia [26]. Recently, Xia Xu found that overexpression of p53R2 and RRM2 could selectively induce the occurrence of lung cancer in transgenic mice. [27]. Abid et al. reported that activated ribonucleotide reductase could increase the production of deoxyribonucleotide triphosphate and induce cell division. The functional activation or overexpression of p53R2 as ribonucleotide reductase leads to the tumorigenicity or cell division via the p53 signaling pathway in tumors of p53 wild-type [28]. Overexpression of the p53R2 protein affects the DNA repair regulated by the p53 gene, Increased base error insertion, increased risk of mutation, leading to genome instability and induce tumorigenesis [29]. Genetic polymorphisms can affect gene expression, enzyme function, protein-to-environment interactions, and risk of susceptible to carcinoma [30]. ZongLin Deng^,^s reported newly identified polym-orphisms in the p53R2 gene,It was found that the 3 ‘end of the p53R2 gene had three gene polymorphisms and one gene polymorphism at the 5’ end, which could increase the risk of carcinoma [31].Before the formation of cancer cells, p53R2 provides dNTPs for DNA repair and increases the expression of p21while decreasing the expression of cyclin D in wild-type p53 cell to arrest cell cycle in order to repair damaged DNA. After the formation of malignancy, their increasing demands for nutrients and support, p53R2 may contribute to cancer cell progression especially when p21 presents in cytoplasm [32]. However, the mechanism of p53R2 protein development in LSC is unclear. Radiosensitivity of ESCC cell lines has been improved by the inhibition of siRNA for p53R2 [33]. Tumor growth is suppressed and sensitivity of 5-FU is increased in oral cancer cells by p53R2 RNA-interference [34]. Knocking down p53R2 of LNCaP cells could block DNA repair and also inhibited the induction of p21 [35]. Exploring the molecular pathway mechanism will be a meaningful attempt. The above evidence showed that p53R2 expression plays a critical role in the occurrence and prognosis of tumors. P53R2 small molecule inhibitors may open a new chapter in the effective treatment of cancer for the clinical development and application of anti-cancer drugs.

In summary, overexpression of p53R2 is associated with poor prognosis in LSC. the expression of p53R2 protein can be used as an independent prognostic factor for assessing the overall survival time of patients with LSC, suggesting that p53R2 protein plays an important role in the development and progression of LSC and is expected to be a potential target for the treatment of LSC.

## Conclusions

The expression of p53R2 was associated closely with the development and progression of LSC, indicated that the presence of p53R2 was an independent factor for poor prognosis of patients with LSC.

## Abbreviations

LSC: Lung sarcomatoid carcinoma
NSCLCs: Non-small cell lung cancer
P53R2: p53-inducible RR small subunit 2 homologue
RR: Ribonucleotide reductase
